# Actinomycetoma Caused by *Actinomadura mexicana*, A Neglected Entity in the Caribbean

**DOI:** 10.3201/eid2602.191005

**Published:** 2020-02

**Authors:** Simon Bessis, Latifa Noussair, Veronica Rodriguez-Nava, Camille Jousset, Clara Duran, Alina Beresteanu, Morgan Matt, Benjamin Davido, Robert Carlier, Emmanuelle Bergeron, Pierre-Edouard Fournier, Jean Louis Herrmann, Aurélien Dinh

**Affiliations:** Hôpital Universitaire Raymond-Poincaré, Assistance Publique–Hôpitaux de Paris, Garches, France (S. Bessis, L. Noussair, C. Jousset, C. Duran, A. Beresteanu, M. Matt, B. Davido, R. Carlier, J.L. Herrmann, A. Dinh);; Claude Bernard University–Lyon I, Lyon, France (V. Rodriguez-Nava, E. Bergeron);; Aix-Marseille University, Marseille, France (P.-E. Fournier);; Institut Hospitalo-Universitaire Méditerranée Infection, Marseille (P.-E. Fournier);; Paris-Saclay University, Versailles, France (J.L. Herrmann)

**Keywords:** *Actinomadura mexicana*, actinomycetoma, antimicrobial susceptibility, bacteria, Caribbean, fungi, gene sequencing, mycetoma

## Abstract

Mycetoma is a chronic infection that is slow to develop and heal. It can be caused by fungi (eumycetoma) or bacteria (actinomycetoma). We describe a case of actinomycetoma caused by *Actinomadura mexicana* in the Caribbean region.

Mycetoma is a neglected tropical disease that poses a major public health problem (*1*). It is endemic in arid or semiarid regions, such as part of the Indian subcontinent, East and West Africa, and Central and South America (*2*). Mycetoma when caused by bacteria is called actinomycetoma; when caused by fungi, eumycetoma. The pathogens are found in the environment, often in soil, and usually infect people through minor or undetected trauma, thorn pricks being the most common (*3*). Bacteria of the *Actinomadura* genus can cause actinomycetoma; the most frequently clinically isolated species are *A. madurae* and *A. pelletieri* (*1*). We report infection with *A. mexicana* that was acquired in the Caribbean.

A 38-year-old woman from Haiti who had arrived in France with no apparent medical problems, was hospitalized a month after her arrival for treatment of multinodular lesions of the left foot and the distal part of the left leg (Figure, panel A). She was afebrile but had multiple bulbous nodules of the foot associated with a nodular lesion. The nodules had central pinpoint ulcerations with granular discharge. The woman was experiencing pain and a complete loss of function of her left foot, symptoms that had been evolving for ≈6 months. 

**Figure:**
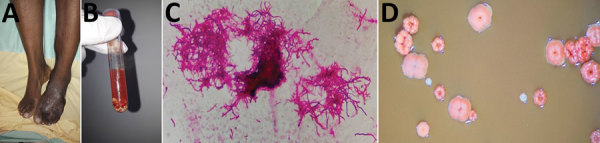
Actinomycetoma caused by *Actinomadura mexicana* infection in a 38-year-old woman from Haiti, France. A) Multinodular lesions on the dorsal surface of the left foot. B) Liquid from puncture of the nodules, showing white-yellow grains. C) Gram-positive bacilli branching out, characteristic of actinomycetal bacteria (original magnification ×1,000). D) Colonies after an 8-day incubation, showing warty ornamentation, pink to pallid red, convex, with a wrinkled morphology (observed through binocular magnifying glass).

Standard radiographs, a computed tomography scan, and magnetic resonance imaging of the affected foot showed symmetrical para-articular marginal erosion in the third and fourth metatarsophalangeal joints, with local inflammation and multiple subcutaneous nodular lesions containing small low-signal foci. A negative result from an HIV serology test and the absence of lymphopenia (2.19 g/L) and hypogammaglobulinemia indicated that there was no immunosuppression. An inflammatory syndrome, with a C-reactive protein level of 53.93 mg/L and a total leukocyte count of 4.5 g/L (neutrophils 1.7 g/L), was identified. 

A sample of the nodules, taken from a punch biopsy, revealed a liquid serum containing white-yellow grains (Figure, panel B). Direct examination showed numerous branching gram-positive bacilli, characteristic of actinomycetal bacteria (Figure, panel C). Histologic analysis revealed abundant filamentous structures consistent with aerobic actinomycetes. Results from Grocott’s methenamine silver staining and Zhiel-Neelssen staining tests were negative at direct examination for mycobacteria and fungus. 

We cultured biopsy specimens on Columbia blood agar in an aerobic atmosphere using chocolate Polyvitex agar under 5% CO_2_ and Sabouraud and Lowenstein media. After an 8-day incubation, the cultures yielded positive results for bacterial colonies, which were pink to red in color and convex with a wrinkled morphology in shape (Figure, panel D). Aerobic and anaerobic blood vials remained negative. A surgical bone biopsy was also performed, and direct examination showed gram-positive branching filaments. Final identification was confirmed by 16S rRNA gene sequencing, as described by Rodriguez-Nava et al. (*4*), using BLAST (https://blast.ncbi.nlm.nih.gov/Blast.cgi) to compare the identified sequence with existing sequences in the GenBank database. The sequence matched >95% with *A. mexicana* (GenBank accession no. MN684846). 

Antimicrobial susceptibility testing, performed using the agar disk diffusion method (Bio-Rad, https://www.bio-rad.com) according to French Microbiology Society guidelines (*5*), showed susceptibility to amoxicillin, amoxicillin/clavulanate, carbapenems, third-generation cephalosporins, aminosides, cyclines, erythromycin, linezolid, vancomycin, sulfamethoxazole/trimethoprim, fluoroquinolones, and rifampin. The patient was given amoxicillin/clavulanate (2 g 3×/d) and sulfamethoxazole/trimethoprim (1,600 mg/280 mg 3×/d) for a minimum of 6 months. At her 3-month follow-up, the woman reported reduced pain and doctors found a decrease in the size of the subcutaneous nodules; magnetic resonance imaging confirmed decreased nodule size and indicated no extension of bone damage. No debridement surgery was performed. 

The patient used to live in a rural village near the town of Gonaïves in the Artibonite district of Haiti, a semiarid and hot region compatible with actinomycetoma (*6*), and she mainly walked barefoot or with open shoes, which may explain her exposure to the bacteria. However, no previous case of actinomycetoma caused by *A. mexicana* had been reported in that area. *A. mexicana* was described by Quintana et al. (*7*) and was isolated with *A. meyerii* from garden soil samples in Mexico in 2003, but a study by Bonifaz et al. published in 2014 found that this species was not identified as a cause of any of the 482 cases of mycetoma recorded in the country during 1980–2013 (*8*). *A. mexicana* was also not identified as the cause of any mycetoma cases reported during 1991–2014 in Brazil (*9*). We could find no accounts in the literature of actinomycetoma in the Caribbean region. The only clinical case of mycetoma found, described by Gugnani and Denning in 2016 (*10*), involved eumycetoma, with etiologic agents such as chromoblastomycoses and *Microsporum canis*. In that article, 2 infections were identified as mycetomas based on case reports in which no laboratory-confirmed microbiological identifications were reported. However, the absence of previous identification might be explained, in part, by lack of access to current molecular biology resources (e.g., matrix-assisted laser desorption/ionization time-of-flight mass spectrometry or PCR). 

This case highlights that actinomycetoma may be present but underrecognized in the Caribbean. Because of the severity of mycetoma and the potential for major socioeconomic effects, healthcare providers in this region should remain informed about the potential risk for these infections. 
